# High grade endometrioid carcinoma arising from deep infiltrating endometriosis extending deeply into the pelvis: a case report

**DOI:** 10.1093/jscr/rjaf266

**Published:** 2025-05-23

**Authors:** Tomohiro Goda, Yasuhiro Yokoyama, Yasumasa Sato, Tomoko Kanda, Mariko Suzuki, Kazuki Sato, Takeaki Saitake, Masaki Katayama

**Affiliations:** Department of Obstetrics and Gynecology, Gifu Prefectural General Medical Center, 4-6-1 Noishiki, Gifu 500-8717, Japan; Department of Obstetrics and Gynecology, Gifu Prefectural General Medical Center, 4-6-1 Noishiki, Gifu 500-8717, Japan; Department of Obstetrics and Gynecology, Gifu Prefectural General Medical Center, 4-6-1 Noishiki, Gifu 500-8717, Japan; Department of Obstetrics and Gynecology, Gifu Prefectural General Medical Center, 4-6-1 Noishiki, Gifu 500-8717, Japan; Department of Obstetrics and Gynecology, Gifu Prefectural General Medical Center, 4-6-1 Noishiki, Gifu 500-8717, Japan; Department of Obstetrics and Gynecology, Gifu Prefectural General Medical Center, 4-6-1 Noishiki, Gifu 500-8717, Japan; Department of Obstetrics and Gynecology, Gifu Prefectural General Medical Center, 4-6-1 Noishiki, Gifu 500-8717, Japan; Department of Pathology, Gifu Prefectural General Medical Center, 4-6-1 Noishiki, Gifu 500-8717, Japan

**Keywords:** carcinoma, endometriosis, chemoradiation, radiation

## Abstract

Carcinoma arising from deep infiltrating endometriosis (DIE) and extending into the rectovaginal septum is extremely rare. Its treatment requires extensive surgical resection and adjuvant chemotherapy. Herein, we present a case demonstrating that concurrent chemoradiation therapy (CCRT) can achieve effective cancer control. A 44-year-old nulliparous woman had been taking oral contraceptives due to pelvic endometriosis for years. The tumor markers have increased, and the tumor was localized within the pelvic cavity, particularly around the vesicouterine pouch and the rectouterine pouch. The patient underwent extensive surgery, but resection was incomplete. Considerable amounts of cancer tissue were retained in the left pelvic wall. CCRT was chosen as an adjuvant therapy due to localized high-grade adenocarcinoma, resulting in a complete response. Treatments for the malignant transformation of extra-gonadal endometriosis vary depending on the involved organs and the degree of extension. CCRT could be selected if the lesion is localized.

## Introduction

Endometriosis can be divided into three phenotypes based on clinical presentation peritoneal/superficial endometriosis, ovarian endometriosis, and deep infiltrating endometriosis (DIE) [1]. It preferentially expands in the pouch of Douglas and may further infiltrate the uterosacral ligaments, torus uterinum, cardinal ligaments, ureters, and bladder, with a particular tendency to involve the rectovaginal septum [2]. Although malignant transformation of DIE is extremely rare, when it does occur, the cancers often invade deeply into adjacent organs, resulting in a poor prognosis.

Herein, we report a case of endometrioid adenocarcinoma that arose from DIE. In this case, effective control was achieved with adjuvant concurrent chemoradiation therapy (CCRT) following incomplete surgical resection. Written informed consent was obtained from the patient for publication of the case report.

## Case presentation

A 44-year-old nulliparous woman with a history of oral contraceptive use due to pelvic endometriosis for years at another hospital. She was referred to our hospital due to elevated levels of carcinoembryonic antigen (41.5 ng/ml) and CA125 (146 U/ml). The gastrointestinal tract was screened and found to be negative for malignancy.

**Figure 1:**
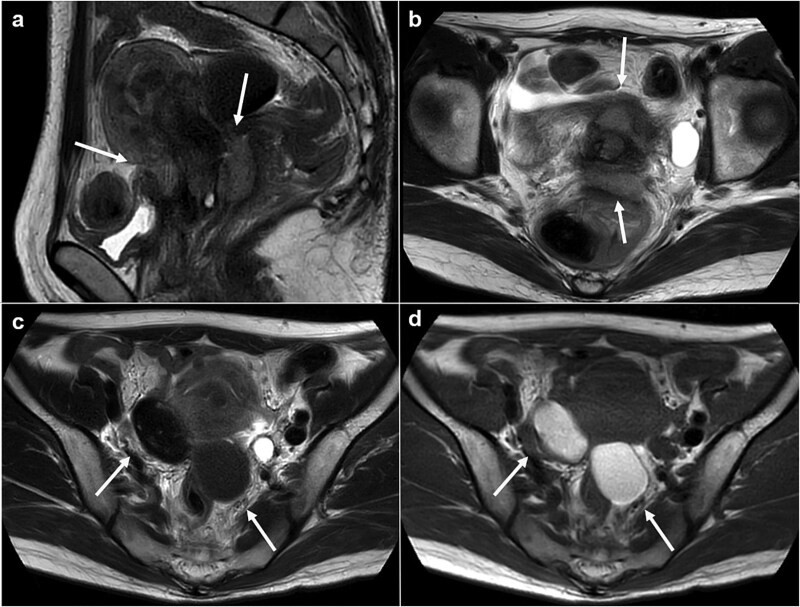
Magnetic resonance images. (a) Sagittal T2 weighted image. (b) Axial T2 weighted image. (c) Axial T1 weighed image. (d) Axial T2 weighed image. Tumors are observed at either the anterior or posterior portion of the urine cervix (a, b: arrows). Ovarian endometriosis is observed in both ovaries (c, d: arrows). Walls of the cysts are smooth.

**Figure 2:**
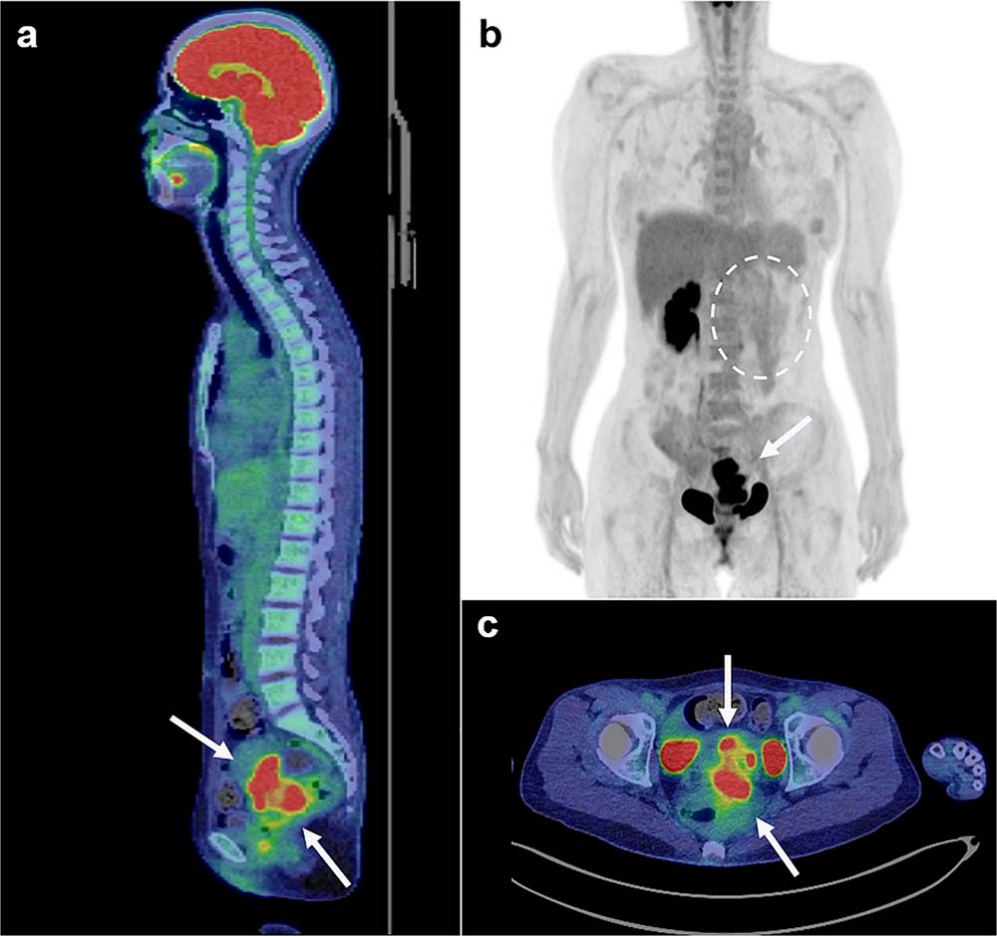
FDG positron emission tomography and computed tomography scans (FDG PET/CT). FDG PET/CT revealed that the mass that had FDG uptake solely in the pelvis (a, b, c: arrows). The left kidney did not uptake FDG because it was non-functioning (b: circle with dotted line).

**Figure 3:**
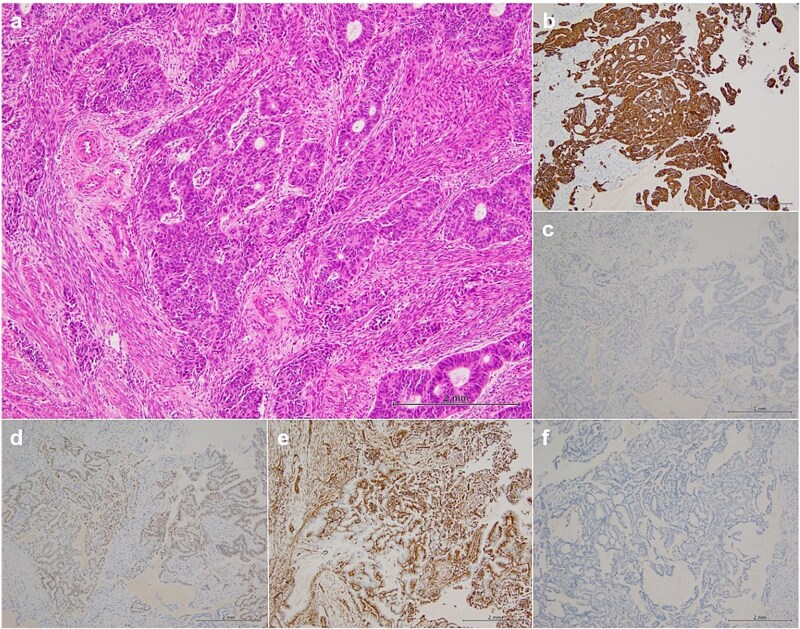
Histopathological findings. Hematoxylin–eosin staining (a) revealed that the histological type was endometrioid carcinoma, grade 3, immunohistochemical staining (b–f) showed positive results for CD7 (b), PAX-8 (d), and ER (e) and negative results for CD20 (c) and CDX-2 (f).

Magnetic resonance imaging revealed masses on the anterior uterine wall, as well as on the posterior uterine wall (Fig. 1a and b). Endometrial cysts were confirmed in the Douglas pouch (Fig. 1c and d). Computed tomography revealed left hydroureter and hydronephrosis, and no distant or lymph node metastases were observed. A Tc99m-diethylenetriaminepentaacetic acid renal renogram showed that the left kidney was nonfunctional. ^18^F-fluorodeoxyglucose (FDG)-positron emission tomography and computed tomography showed that abnormal FDG accumulation was observed only in the pelvis (Fig. 2). At first, no lesions were found on the uterine cervix or vaginal walls, and the cervical and endometrial cytology were negative. However, as time passed, the tumor emerged on the posterior vaginal fornix and was histologically diagnosed as high-grade endometrioid carcinoma (Fig. 3).

**Figure 4:**
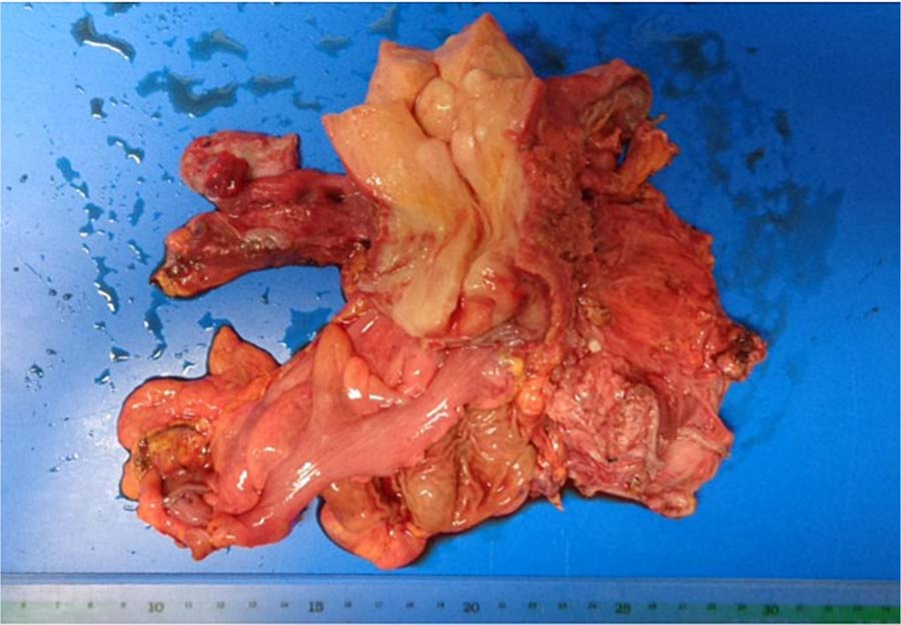
Surgical specimen. The rectum and left ureter were resected together with the uterus.

At the risk of traumatic injury, a stent was placed in the right ureter before surgery. Laparotomy revealed endometrial cysts in the bilateral ovaries. The peritoneum was found to be free from dissemination of the cancer, and the tumor had grown beneath the peritoneum to infiltrate the rectovaginal septum, the uterus, the vagina, the rectum, the parametrium including the bilateral cardinal ligaments, the ureters, and the bladder. Tumor invasion was more extensive on the left side. Carcinoma appeared to infiltrate into the bladder as well as the lower segment of the right ureter, but separation of the bladder and the ureter from carcinoma tissue was barely feasible. Hysterectomy, partial vaginectomy, bilateral salpingo-oophorectomy, partial rectal resection and left ureterectomy, omentectomy, and colostomy were performed (Fig. 4). However, the surgical resection was incomplete, and considerable amounts of cancer tissue were retained, especially in the left pelvic wall.

The histological investigation revealed that there were both endometriosis lesions and cancer present, and carcinoma. Carcinoma was not found in the omentum, the ovaries (including the endometrial cysts), or biopsied peritoneum. Because there was no continuity with the ovarian endometriotic cyst, the pathological investigation concluded that the carcinoma had originated from DIE beneath the peritoneum of the vesico-uterine pouch and/or Douglas pouch, and invaded the connective tissues surrounding the uterine cervix and subsequently extended into the rectovaginal septum, the rectum, the vagina, the bladder, and the ureters.

Because the tumors were located solely in the pelvis, CCRT using cisplatin and 5-fluorouracil was chosen as adjuvant therapy. The colostomy was closed at 10 months, and the patient survived without evidence of recurrence at 24 months after the primary surgery.

## Discussion

Endometriosis is a benign gynecological disease that affects women of reproductive age. Malignant transformation of endometriosis is relatively rare and occurs in ~0.7%–1.0% of patients with endometriosis [3]. The mechanism of malignant transformation is largely unknown. Some studies have proposed a possible mechanism by which excessive hemoglobin, heme, and iron could cause an imbalance in intracellular redox homeostasis in endometriotic cells [4] that induce alterations in cancer driver genes such as TP53, KRAS, PTEN, PIK3CA, and ARID1A [5]. The diagnostic criteria for the malignant transformation of endometriosis include the classifications by Sampson and Scott [6–8].

Endometriosis, especially DIE, may cause ureteral stenosis; however, it rarely provokes renal failure. In this case, the left kidney totally lost its function at diagnosis, suggesting that the insidious progression of carcinoma and direct invasion into the ureter is associated with renal failure. Parametrial invasion with renal failure from carcinoma arising from endometriosis has never been found in previous studies. For such a locally advanced disease, chemotherapy and/or irradiation rather than surgery may be a primary treatment option. However, in this patient, exploratory laparotomy was essential for diagnosing and determining subsequent treatment. On laparotomy, we recognized that complete resection of carcinoma was not feasible and that chemoradiation was a choice as a post-operative treatment.

DIE-originating carcinomas extending into the rectovaginal septum have only been reported in a limited number of past reports in the literature [9–14]. In all reported cases, extensive surgical resection including the rectum and the uterus, and adjuvant therapy were performed. Chemotherapy was selected as adjuvant therapy in most cases, and irradiation was exceptionally utilized.

Yazbeck *et al.* utilized chemotherapy as preoperative treatment [13]. While, Ulrich *et al.* utilized it postoperatively, however, patients suffered a recurrence in one of the two cases [14]. In gastrointestinal stromal tumors developing at this site, chemoradiation is the most commonly used therapy [15]. However, DIE-originating carcinoma is poorly defined.

Endometriosis typically occurs in the ovaries and the pelvic peritoneum. The malignant transformation from these sites is treated as ovarian or peritoneal cancer. Standard treatment includes surgical resection and adjuvant chemotherapy. Even in cases confined to the pelvis, carcinomas are prone to disseminate into the peritoneum. Thus, chemotherapy would be a better choice as adjuvant therapy. In this case, however, carcinoma had developed beneath the peritoneum and extended deeply toward the pelvic floor as well as the lateral pelvic wall. Because the lesion was supposedly confined to the pelvis, it was assumed that chemoradiation would be more promising than chemotherapy in this case.
